# Post-approval Activities Providing Data on the Safety of Medication Use During Pregnancy and Lactation—A TransCelerate Perspective

**DOI:** 10.1007/s43441-025-00764-4

**Published:** 2025-03-16

**Authors:** Maria Fernanda Scantamburlo Fernandes, Amalia Alexe, Olatayo Apara, Lindsey Force, Christine Taeter, Maria Weber, Keele Wurst, Nadezda Abramova, Anju Garg, Leesha Balramsingh-Harry, Jessica Mårlind Würtele

**Affiliations:** 1 Eli Lilly, Indianapolis, USA; 2 Novartis, Basel, Switzerland; 3 Merck & Co. Inc, Rahway, USA; 4 Gilead Sciences Inc, Foster City, USA; 5 UCB, Brussels, Belgium; 6 Boehringer Ingelheim International, Ingelheim, Germany; 7 GlaxoSmithKline, London, UK; 8 Merck Healthcare KGaA, Darmstadt, Germany; 9 Sanofi, Paris, France; 10 Genentech Hoffman LaRoche Ltd, South San Francisco, USA; 11 AstraZeneca, Cambridge, UK

**Keywords:** Post-approval safety activities, Post-approval surveillance, Medication use, Pregnancy, Lactation, TransCelerate survey

## Abstract

Pregnant and lactating women are frequently excluded from clinical trials, leading to a significant global unmet need for safety data regarding medication use in this population. Post-approval safety activities on pregnancy and lactation are currently the main sources of information for product labeling to guide clinical practice. However, generating this information can take years, and the data often remains insufficient for healthcare providers and patients to make informed decisions. Given the differences in regulatory guidance on this issue and the evolving perspectives on the most appropriate types of post-approval activities on pregnancy and lactation, TransCelerate BioPharma conducted a survey of its member pharmaceutical companies to evaluate common post-approval practices over the past 11 years. All survey participants reported engaging in post-approval activities on pregnancy, citing pregnancy registries as the most common type of activity, followed by database studies and enhanced pharmacovigilance. These activities resulted in outcomes, including updates to the prescribing information, however these materialized after many years. Conversely, fewer post-approval activities on lactation were conducted, with limited impact on outcomes reported to date. These results emphasize the need for a comprehensive, multi-faceted approach using a wide array of data sources for effective and timely post-approval surveillance to characterize medication use during pregnancy and lactation.

## Introduction

When medicinal products are approved and first enter the market, there is often limited or no human data about their safety during pregnancy or lactation. During clinical development of new medicines, pregnant and lactating women are typically excluded from clinical trials, thereby leading to a lack of evidence-based information for healthcare professionals to use when prescribing or counseling pregnant women [1]. As a result, fewer than 10% of medicines approved since 1980 have sufficient information to assess their safety during pregnancy [2]. Similarly, only 16% of labels include information on lactation from human data [3]. Nevertheless, studies indicate that between 44 and 99% of pregnant women use at least one prescription medication during their pregnancy [4, 5].

Post-approval pregnancy and lactation safety activities are currently the main sources of information for product labeling to guide clinical practice (Table 1). This information may take years to generate and, many times, these activities are still insufficient to provide healthcare providers and patients with information to make an informed decision. Thus, information to guide decision-making for safe and effective use of medications during pregnancy and lactation is a significant unmet medical need that hinders maternal and child healthcare [6, 7].

**Table 1.** In-Scope Post-approval Activities (Company-Sponsored) Focused on Data Generation in Pregnancy.

Pregnancy registry	Observational/non-interventional cohort study with or without a comparison group including pregnancy sub-studies in product or disease registries
Database study	Non-interventional study with or without a comparison group that utilizes electronic administrative claims databases and/or EHR or other data to assess pregnancy outcomes Note: For purposes of the survey, responding companies were asked to consider a study that uses a registry as data source under the first category: pregnancy registry
Enhanced pharmacovigilance (PV)	Enhanced follow-up activities or data collection for specific product(s) in addition to what is routinely performed for any spontaneous reports related to pregnancy received by a company. For example, there may be additional time points of follow-up (i.e., subset of products for which follow-up is performed at 6 months after birth and 1 year after birth) or specific data collection form used for specific product(s)
Interventional study	Clinical trial or pharmacokinetic (PK) study with or without comparison groups in pregnant women
Drug utilization study	Study to determine product use in pregnancy to inform on further work (e.g. pregnancy registry, database study, interventional study)

Post-approval safety activities play a crucial role in the collection of valuable safety information following drug use outside the well-controlled environment of clinical trials. The increased patient exposure in real-world settings after marketing authorization (MA) allows for a more comprehensive evaluation of a drug’s safety profile (particularly in populations that are not studied during clinical development) and aids in the identification of rare or very rare adverse events. Most information on medication use during pregnancy and lactation comes from observational data in the post-approval setting. There are various types of post-approval observational activities that can provide information on medicine exposure during pregnancy and lactation. However, there is a need to improve the timeliness and quality of these activities so that they yield as much relevant information as possible.

A regulatory landscape assessment performed by TransCelerate BioPharma (a non-profit organization that aims to collaborate across the global biopharmaceutical community and improve the research and development of new therapies) [8] showed that as of May 2022, there was no globally accepted International Council for Harmonisation of Technical Requirements for Pharmaceuticals for Human Use (ICH) standard to guide the conduct of post-approval pregnancy and lactation safety activities, and there were no globally harmonized regulations or guidance on post-approval pregnancy safety activities. Furthermore, there have been no significant updates in the regulations on this topic to date.

While regulatory guidances exist in the area of post-approval safety activities, they vary depending on the ruling Health Authority (HA). For example, the European Medicines Agency (EMA) provides some guidance on considerations for additional pharmacovigilance (PV) studies [9]. Potential types of activities to generate safety evidence in pregnancy and lactation include all epidemiological designs in principle, including (but not limited to) pregnancy registries [9]. The European Union (EU) guidance recommends that, where feasible, epidemiological studies should be carried out using existing data sources (i.e., secondary database studies) and be designed in such a way to minimize bias and confounding [9]. The EU guidance also calls for Sponsors to consider the use of existing pregnancy registries or databases to enhance long-term follow-up, facilitate the inclusion of comparator groups, and make use of existing infrastructure for data collection and analysis to avoid unnecessary duplication of effort as well as enhance overall efficiency [9]. Additionally, the EMA considers registries that aim to capture all pregnant women with a specific disease (disease-based pregnancy registry) as generally more useful than medicinal product-specific registries (drug pregnancy registry) because they provide information longitudinally throughout pregnancy, allowing potential comparisons between products and pregnancy outcomes in an unexposed population [9].

The US Food and Drug Administration (FDA) guidance generally considers the use of secondary electronic data sources (e.g., insurance claims and electronic health record [EHR] databases), population-based surveillance, and national registries/registers or population-based case control studies as additional studies that complement data obtained from pregnancy registries [10]. In fact, FDA guidance requires a pregnancy exposure registry to be seriously considered when it is likely that the medical product will be used during pregnancy as therapy for a new or chronic condition [11]. Both the FDA and EMA have publicly highlighted some of the limitations of pregnancy registries, including the failure to provide clinically meaningful information because of inadequate enrollment [12] and obtainment of quality pregnancy exposure data, potential selection bias due to the voluntary nature of patient enrollment, and loss to follow-up [13].

In 2023, the FDA held a public workshop, where current processes to collect and evaluate post-approval pregnancy safety information were assessed and a draft framework that provided more flexibility in the type of design of a post-approval activity was introduced [14]. The framework allows for the advantages and disadvantages of various types of activities to be considered. It also considers the number of exposed pregnancies expected, which may differ for individual diseases and medications. The workshop highlighted that a variety of data sources could be utilized to monitor medication use during pregnancy, and potentially provide information on human pregnancy. However, there is variability in the utility of different activities to inform product labeling.

Considering the differences in regulatory guidances [15] and the evolving views on utilizing the most appropriate post-approval activities on pregnancy and lactation based on disease and medicinal product characteristics, the Pregnancy & Breastfeeding team within the TransCelerate Interpretation of Pharmacovigilance Guidances & Regulations workstream took the initiative to explore practices among pharmaceutical companies over the last 11 years. The team conducted a survey among the TransCelerate BioPharma member companies to assess the types of post-approval activities pharmaceutical companies had performed, the type of information arising from these activities, and the impact on prescribing information (PI) updates and other risk minimization activities.

## Methods

The Pregnancy & Breastfeeding team within the TransCelerate Interpretation of Pharmacovigilance Guidances & Regulations workstream conducted a survey among 17 TransCelerate BioPharma member pharmaceutical companies from April 2024 through May 2024. The survey was sent to 21 TransCelerate BioPharma member pharmaceutical companies. Survey participants (each representing a member company) were asked to provide information on the type, number, and data collection format of post-approval activities focused on data generation in pregnancy and lactation that were started, ongoing, or completed between January 1, 2013, and December 31, 2023. The post-approval activities on pregnancy in the survey’s scope included pregnancy registries, database studies, enhanced PV, interventional studies, and drug utilization studies (Table 1). For lactation, the in-scope post-approval activities included lactating women (milk-only) studies, lactating women (milk and plasma) studies, mother-infant pair studies on lactation, and lactation sub-studies in pregnancy registries (Table 2). Definitions for the post-approval activities on pregnancy and lactation (Tables 1 and 2, respectively) were provided to the survey participants, outlining how to interpret terms in evaluating and framing their responses to the survey questions.

**Table 2.** In-Scope Post-approval Activities (Company-Sponsored) Focused on Data Generation in Lactation.

Lactating women (milk-only) study	Study conducted to detect the presence of a drug in breast milk, quantify or estimate the total amount of a drug transferred into breast milk (when plasma concentrations are known), and evaluate the effects of a drug on milk production (when milk production in lactating women not taking the drug is known)
Lactating women (milk and plasma) study	Milk and plasma collection in lactating women can provide PK data on a drug in a lactating woman, the amount of drug transferred into breast milk, and the effects of a drug on milk production
Mother-infant pair study on lactation	Mother-infant pair study that includes assessment of drug concentrations in infants can provide information on absorption of drugs in infants through breast milk and safety assessments in infants enrolled in these studies
Lactation sub-study in pregnancy registries	Pregnancy registries in which newborns are further observed during exposure via lactation

The survey did not seek information about post-approval activities relating to routine primary data collections in PV (except individual case safety reports [ICSRs] subject to enhanced PV, per definition in Table 1), pregnancy prevention programs, assessment of risk minimization activities, feasibility studies, post-authorization efficacy studies without collection of safety data, vaccine pregnancy exposure registries considered passive surveillance, and activities being performed for scientific reasons by the survey participants as part of their evidence generation plans but not planned for submission to HAs.

Survey participants were asked about the initiator of the post-approval activities (i.e., proposed by the Sponsor/Marketing Authorization Holder [MAH] or requested by HAs) and the context (e.g., initial MA, subsequent regulatory procedure, or post-approval safety signal). Further, the survey asked about the high-level impact of the types of post-approval activities, including the inclusion of data in the PI, modification of the pregnancy recommendation in the PI, changes to the safety concerns in the risk management plan (RMP), development or modification of risk minimization measures (e.g., creation of educational material for patients/prescribers, patient reminder card, or digital healthcare professional communications [DHPC]), initiation of another post-approval activity to generate data, evaluation of a new safety finding, and publication of data.

Data to support quantification were collected according to the number of post-approval activities by type (Tables 1 and 2) with the range categories 0, 1–4, 5–10, and > 10. Information about the post-approval activities generating data on pregnancy and lactation was requested overall across the product portfolio of each survey participant and then separately on pregnancy and on lactation. Survey participants were asked to provide their response in a web-based survey template, with the option of not answering 1 or more questions. A third-party project manager managed the survey, collected all responses from the survey participants, and blinded and aggregated all responses before sharing the responses with the member companies and the Pregnancy & Breastfeeding team. The TransCelerate Pregnancy & Breastfeeding core team then evaluated these data and summarized the results.

### Evaluation Approach

The aggregated survey results were analyzed in a descriptive manner. Regarding the number of post-approval activities focused on pregnancy and/or lactation, the survey asked for ranges rather than unique numbers. Using those ranges, the team calculated the lowest minimum and highest maximum number of post-approval activities possible, given these ranges, which outcomes have been presented in Table 3.

**Table 3:** Minimum and Maximum Number of Post-Approval Activities on Pregnancy (Proposed by Sponsor/MAH and Requested by HAs) and Their Impact Outcomes.

Types of Post-approval Activities	Pregnancy Registry		Database Study		Enhanced PV		Interventional Study		Drug Utilization Study	
Range of Post-approval Activities^a^	Min n = 73	Max^c^ n > 146	Min n = 37	Max n = 98	Min n = 20	Max n = 70	Min n = 8	Max n = 32	Min n = 11	Max n = 34
Impact of post-approval activities^b^										
Inclusion of data in the PI	8	32	4	16	1	4	2	8	1	4
Modification of pregnancy recommendation in the PI	3	12	1	4	1	4	2	8	0	0
Removal of missing information from RMP	3	12	5	20	1	4	0	0	2	8
Reclassification of safety concern to important risk	1	4	0	0	1	4	0	0	0	0
Development or modification of risk minimization measures	2	8	2	8	2	8	0	0	0	0
Initiation of another post-approval activity to generate data	5	20	2	8	2	8	0	0	2	8
Evaluation of new safety finding	4	16	2	8	0	0	1	4	0	0
Poster/abstract/publication	10	30	3	12	4	16	2	8	1	4

^a^Numbers in this table were calculated as follows: Survey participants who either proposed or were requested by HAs to conduct at least 1 post-approval activity on pregnancy were asked to classify the number of their activities into 4 different category ranges (0, 1–4, 5–10, > 10). The count of survey participants reporting at least 1 activity was multiplied by the category range, providing a minimum and a maximum count for each activity by type. Numbers presented in this table combine numbers of Sponsor-proposed activities and HA-requested activities to give total activity counts.

^b^Survey participants were asked if any post-approval activity on pregnancy resulted in a specified impact and to classify these activities into 4 different category ranges (0, 1–4, 5–10, > 10). The count of survey participants reporting at least 1 impact was multiplied by the category range, providing a minimum and a maximum count of activities for each impact type.

^c^The category range > 10 was reported by 2 survey participants for the pregnancy registry activity; therefore, a precise maximum count cannot be provided for this activity.

The content in this Paper is provided for informational purposes only and should not be construed as conveying legal advice. Any party using these materials to determine the regulatory landscape across jurisdictions for purposes of drug development, drug approval, or patients’ safety or any other purposes bears sole and complete responsibility for determining what laws, regulations, and guidances apply to its conduct and operations in each relevant jurisdiction and complying with (including how best to comply with) all applicable laws and regulations in all relevant jurisdictions. The views and opinions expressed herein are those of the authors; they do not necessarily reflect those of their affiliated companies.

## Results

### Overall Results

**Figure 1.**
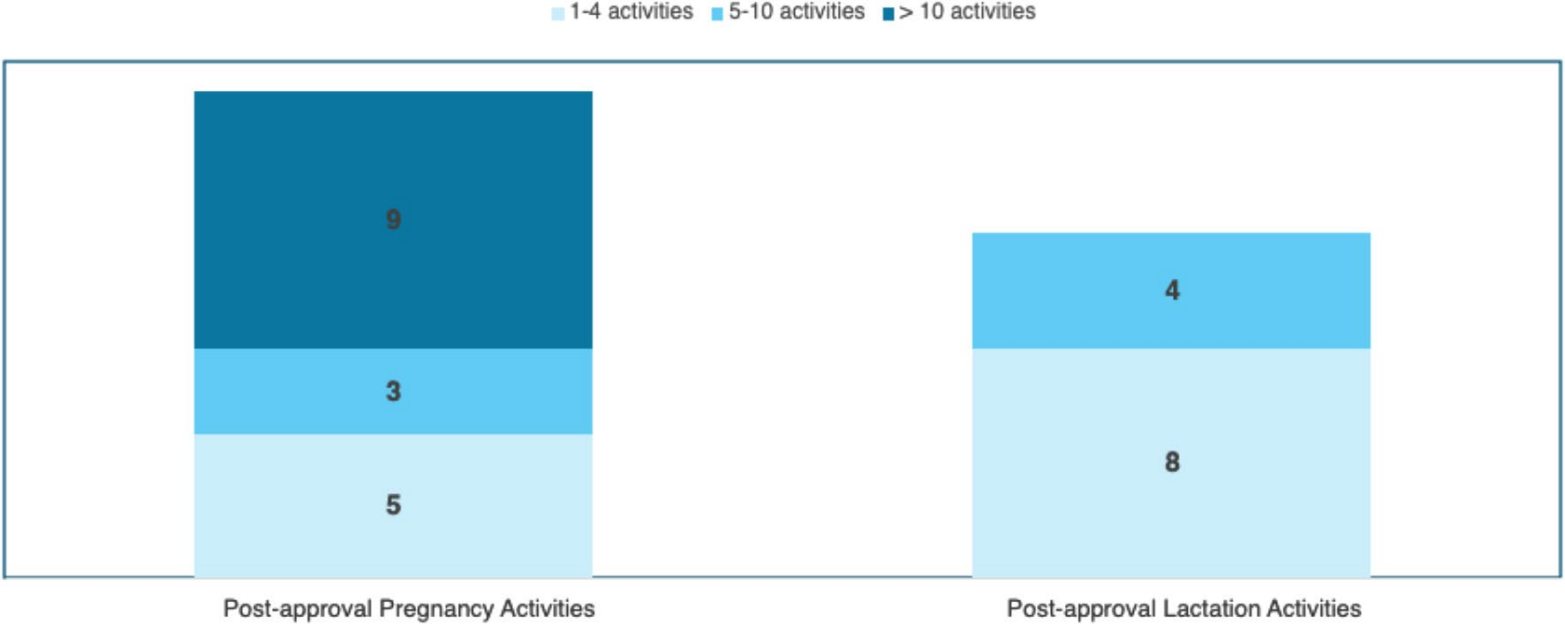
Number of survey participants reporting post-approval activities on pregnancy and/or lactation.

Seventeen (17) of the TransCelerate member companies responded to the survey in whole or in part. Of the 17 survey participants, all reported at least 1 post-approval activity focused on data generation in pregnancy and/or lactation that was under protocol development, submitted, ongoing, or completed within the pre-specified 11 year period (January 1, 2013, to December 31, 2023). Overall, there was a greater number of post-approval activities on pregnancy than on lactation (Fig. 1).

**Figure 2.**
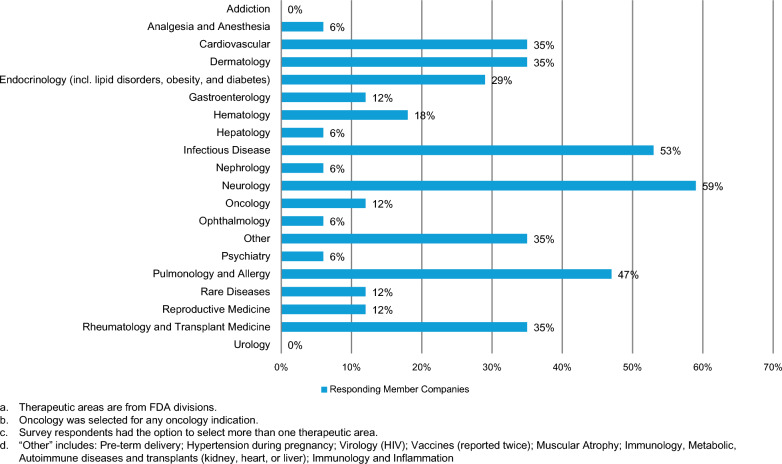
Therapeutic areas with post-approval activities on pregnancy and/or lactation.

The therapeutic areas (more than one could be selected) with the highest proportion of companies reporting post-approval activities on pregnancy and lactation were neurology (59%; 10/17), infectious disease (53%; 9/17), and pulmonology and allergy (47%; 8/17). Figure 2 summarizes all the therapeutic areas with post-approval activities. The product types with regard to which survey respondents conducted the post-approval activities included, among others small molecules, monoclonal antibodies, and proteins and peptides. Additionally, nearly all (88%, 15/17) survey participants had post-approval activities on pregnancy and lactation that were registered in the Heads of Medicines Agencies (HMA)-EMA Catalogue of real-world data (RWD) studies [16] and/or the National Institutes of Health (NIH) National Library of Medicine [17].

**Figure 3.**
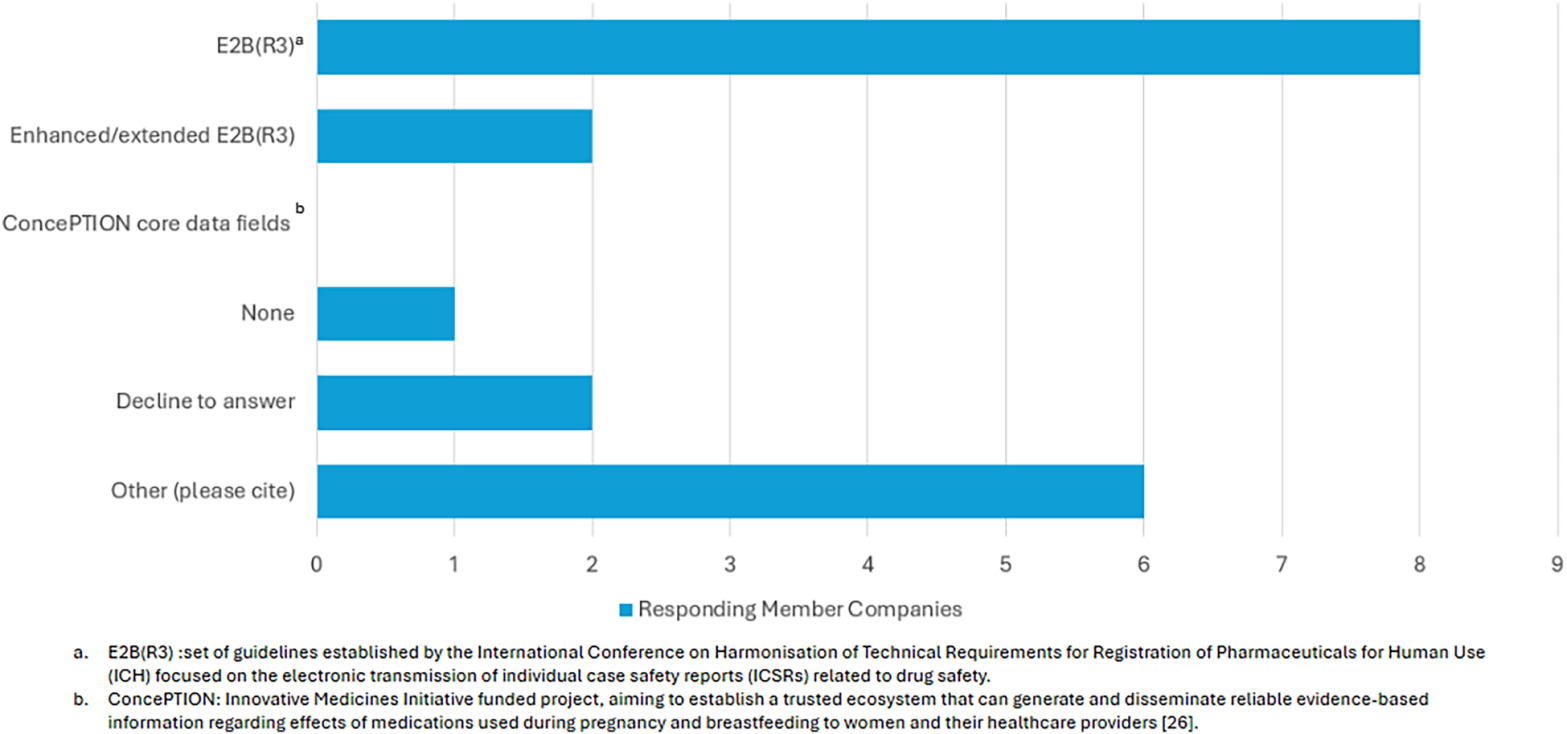
Data format for primary data collection on pregnancy and lactation.

As shown in Fig. 3, most survey participants noted that the E2B(R3).was used for pregnancy and lactation primary data collection; however, other data formats were also utilized. Where “other” was selected by survey participants, the following data formats were cited (responses in free text): E2B R2, Council for International Organizations of Medical Sciences (CIOMS), MedWatch, clinical registries, standard internal case report form (CRF), follow-up forms, modified ConcePTION core data fields [18], and custom standard per study.

### Post-approval Activities Focused on Data Generation in Pregnancy

**Figure 4.**
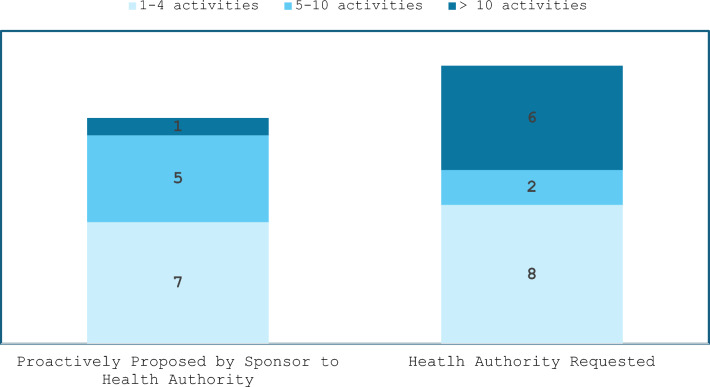
Number of post-approval activities on pregnancy: proposed by Sponsor/MAH vs requested by HAs.

All 17 survey participants conducted post-approval activities on pregnancy. Seventy-six percent (13/17) conducted post-approval activities that were proactively proposed by the Sponsor/MAH to the HAs, whereas 94% (16/17) conducted post-approval activities that were requested by the HAs (Fig. 4; participants could select more than 1 type of initiator).

### Post-approval Activities Proposed by Sponsor/MAH—Types and Reason

**Figure 5.**
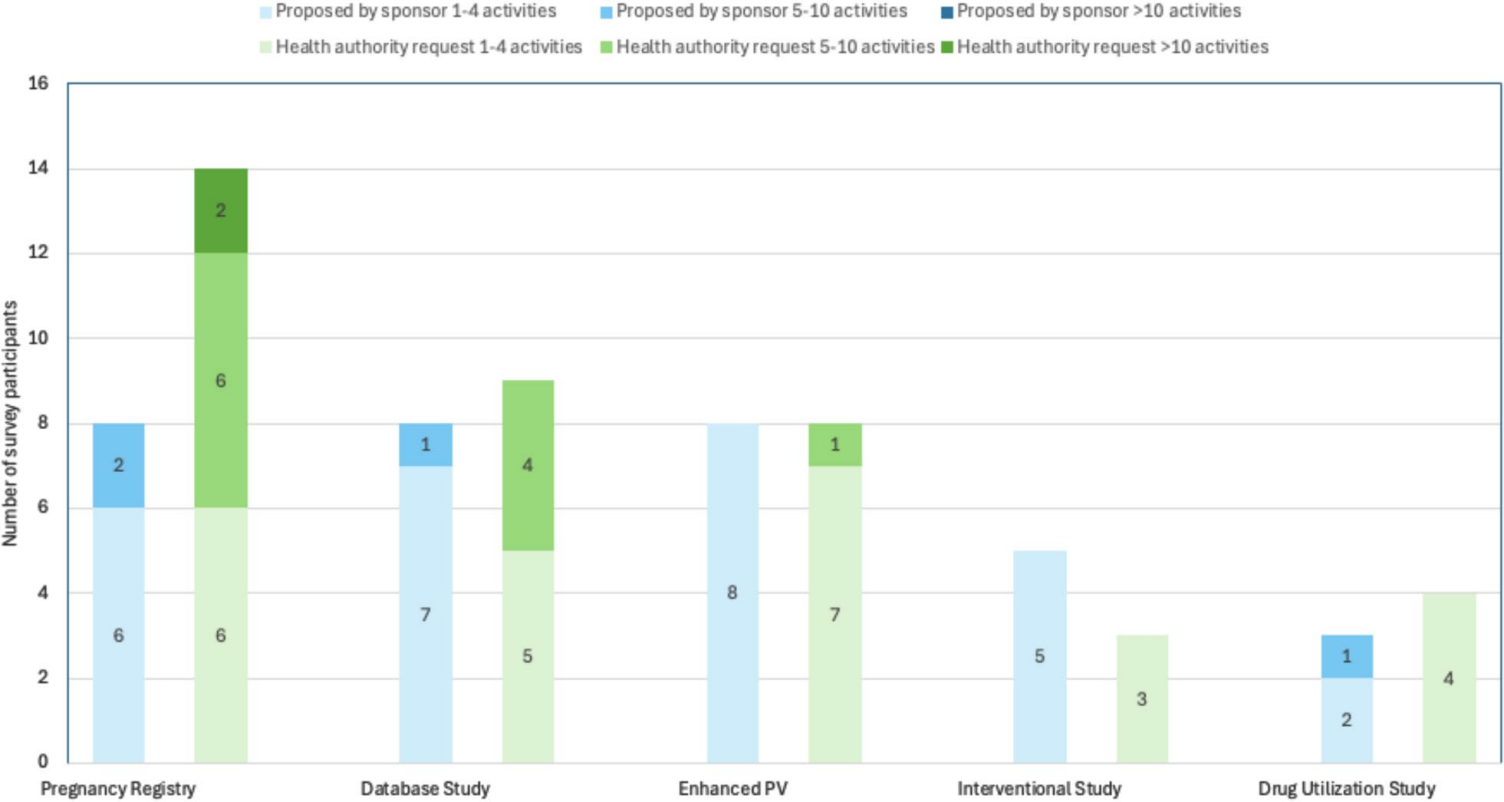
Types of post-approval activities on pregnancy: proposed by Sponsor/MAH vs requested by HAs.

Among the 13 survey participants that reported post-approval activities on pregnancy that were proactively proposed by the Sponsor/MAH to the HAs, 62% (8/13) had at least 1 post-approval activity with the following types: pregnancy registry, database study, or enhanced PV. Thirty-eight percent (5/13) of these survey participants proposed an interventional study, while only 23% (3/13) proposed a drug utilization study (Fig. 5).

**Figure 6.**
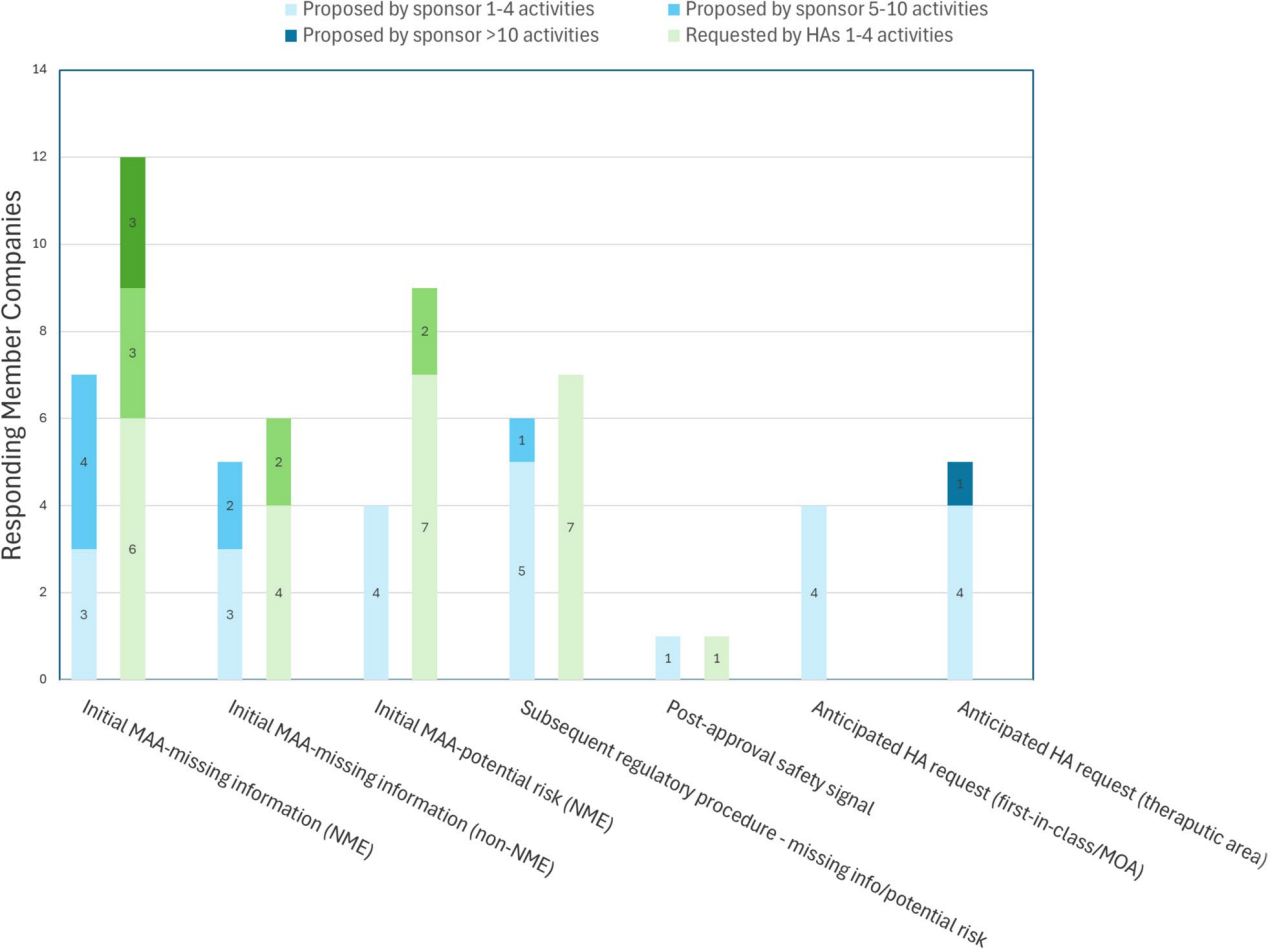
Reason for the post-approval activities on pregnancy: proposed by Sponsor/MAH vs requested by HAs.

In regards to the reason for post-approval activities on pregnancy that were proposed by the Sponsor/MAH to the HAs, the majority of proposals occurred at the initial marketing authorization application (MAA) for a new molecular entity (NME), while 31% (4/13) of the survey participants reported the reason as an anticipated HA request due to being first-in-class/mechanism of action (MOA) and only 8% (1/13) reported the reason to be a post-approval safety signal. Figure 6 summarizes all reported reasons for proposed activities on pregnancy.

### Post-approval Activities Requested by HAs—Types and Reason

Among the 16 survey participants that reported post-approval activities on pregnancy that were requested by HAs, 88% (14/16) indicated that a pregnancy registry was the most frequently requested type of post-approval activity, followed by a database study (56%; 9/16), enhanced PV (50%; 8/16), a drug utilization study (25%; 4/16), and an interventional study (19%; 3/16) (Fig. 5).

The reason for most of the HA-requested activities was to address “missing information” on the use of medicine during pregnancy per Good Pharmacovigilance Practice ( GVP) Module V (75%; 12/16) and potential risks (56%; 9/16) in the safety specification of an NME during the initial MAA, whereas 38% (6/16) of survey participants indicated that the HA request during the initial MAA was due to missing information for a non-NME. Additionally, 44% (7/16) of survey participants indicated that the HA request was at a subsequent regulatory procedure interaction, while only 6% (1/16) indicated that HAs requested the activities to evaluate a post-approval safety signal (Fig. 6). Lastly, 71% (12/17) of all survey participants reported that more than 1 post-approval activity on pregnancy or lactation for at least 1 product was initiated due to varying HA requests for the same product.

### Impact of Post-approval Activities on Pregnancy

**Figure 7.**
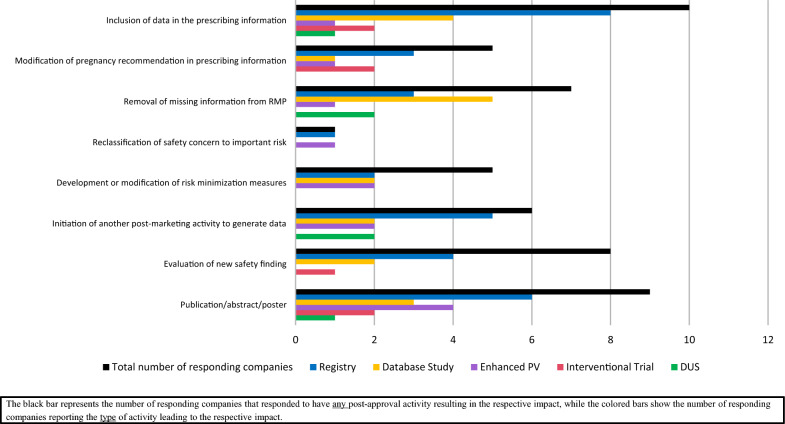
Impact of post-approval activities on pregnancy (proposed by Sponsor/MAH and requested by HAs).

The impact of post-approval activities on pregnancy (proposed by Sponsor/MAH and requested by HAs) by type of impact and activity is summarized in Fig. 7. For 59% (10/17) of all survey participants, data generated by the post-approval activities led to the inclusion of data in the PI, wherein the main sources of pregnancy data were pregnancy registries and database studies. Twenty-nine percent (5/17) of the survey participants reported a modification of pregnancy recommendation in the PI, with 4 participants reporting a loosening of restriction on pregnancy recommendation and 1 reporting an increase in restriction of pregnancy recommendation. The time intervals between initial MA and PI modification (reported by 4 survey participants) were between 5 and 10 years for half of the responding participants and greater than 10 years for the other half of responding participants.

For 41% (7/17) of the survey participants, the post-approval activities on pregnancy led to the removal of missing information from the RMP as per GVP V, while 6% (1/17) reported the reclassification of safety concern to important risk; these outcomes were based on data generated mainly from database studies and pregnancy registries, followed by enhanced PV and drug utilization studies. Twenty-nine percent (5/17) of participants reported development or modification of risk minimization measures (resulting from data generated from pregnancy registries, database studies, and enhanced PV) and 35% (6/17) reported that post-approval activities (mostly pregnancy registries followed by database studies, enhanced PV, and drug utilization studies) led to the initiation of another post-approval activity to generate additional data.

For 47% (8/17) of the survey participants, the post-approval activities resulted in an evaluation of a new safety finding; the most frequent sources of new safety findings were registries followed by database studies and interventional studies. Lastly, 53% (9/17) of all survey participants reported that pregnancy data (from mainly pregnancy registries, followed by enhanced PV and database studies) were published in posters, abstracts, or publications.

To provide numerical context to these impact outcomes, Table 3 summarizes results from multiple survey questions that allowed calculation of the lowest possible minimum and highest possible maximum number of post-approval activities on pregnancy (by type) and of impact outcomes (per post-approval activity) reflected in the survey responses.

### Post-approval Activities Focused on Data Generation in Lactation

**Figure 8.**
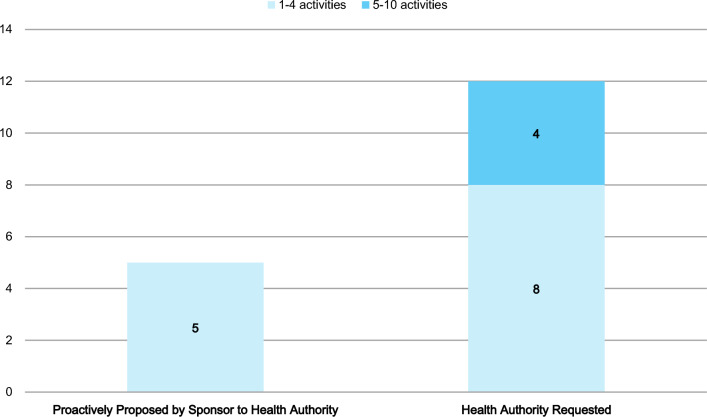
Number of post-approval activities on lactation: proposed by Sponsor/MAH vs requested by HAs.

Seventy-one percent (12/17) of all the survey participants conducted post-approval activities on lactation, of which 42% (5/12) conducted post-approval activities that were proactively proposed by the Sponsor/MAH to the HAs and 100% (12/12) conducted post-approval activities that were requested by HAs (Fig. 8, participants could select more than 1 type of initiator). All 12 survey participants that reported post-approval activities on lactation (encompassing both Sponsor-proposed and HA-requested activities) responded that they conducted somewhere within a range of between 1 and 4 activities.

### Post-approval Activities Proposed by Sponsor/MAH—Types and Reason

**Figure 9.**
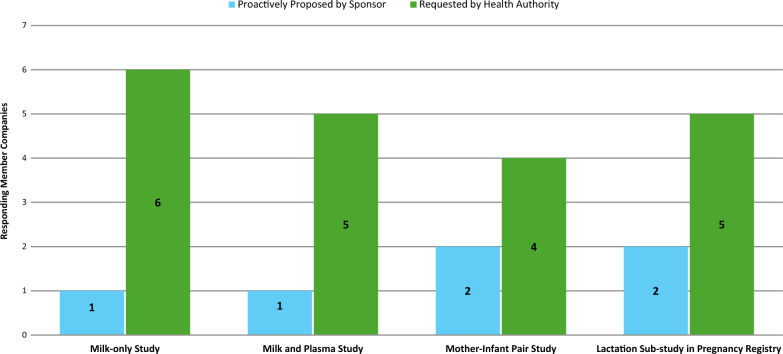
Types of post-approval activities on lactation: proposed by Sponsor/MAH vs requested by HAs.

Among the 5 survey participants that reported post-approval activities on lactation that were proactively proposed by the Sponsor/MAH to the HAs, at least 1 participant conducted 2 types of post-approval activities. Specifically, more survey participants proposed a mother-infant pair study on lactation and a lactation sub-study in pregnancy registries compared to a lactating women (milk-only) study or a lactating women (milk and plasma) study (Fig. 9). The post-approval activities on lactation that were proposed by the Sponsor/MAH were primarily conducted at the beginning of the product life cycle, with the reported reasons being MA submission and first-in-class MA submission.

### Post-approval Activities Requested by HAs—Types and Reason

Among the 12 survey participants that reported post-approval activities on lactation that were requested by HAs, there was a more even spread on the types of activities requested (i.e., lactating women [milk-only] study, lactating women [milk and plasma] study, lactation sub-study in pregnancy registries), with slightly fewer participants reporting a mother-infant pair study on lactation (Fig. 9). The reason for most of the HA-requested activities was to address early-stage requirements of the product life cycle, such as MA submission.

### Impact of Post-approval Activities on Lactation

**Figure 10.**
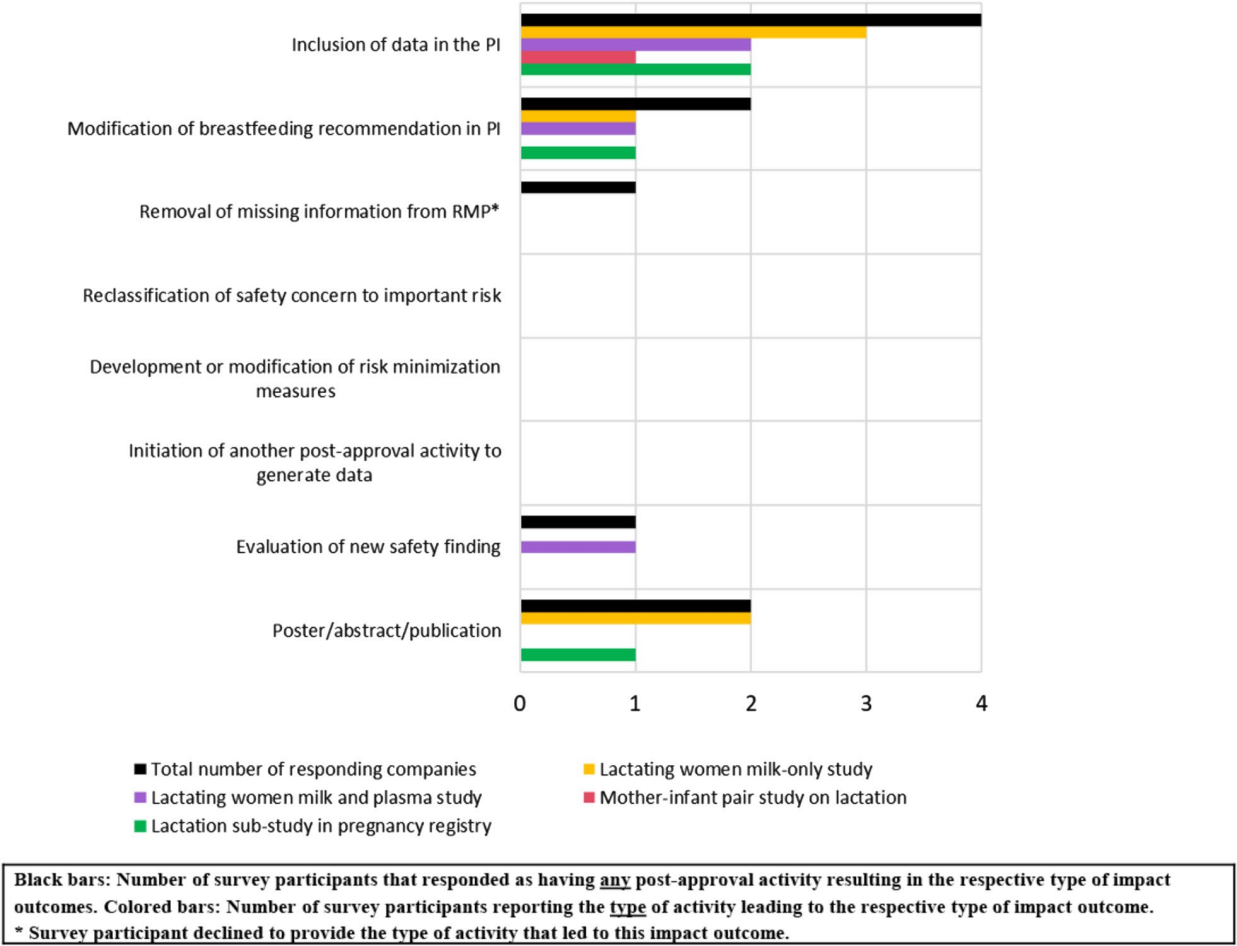
Impact of post-approval activities on lactation (proposed by Sponsor/MAH and requested by HAs).

The impact of post-approval activities on lactation (proposed by Sponsor/MAH and requested by HAs) were reported by 11 survey participants, with only a minority of participants reporting that activities resulted in an actual outcome (each respondent could select more than 1 answer). Most survey participants reported 0 impact outcomes resulting from the lactation activities, regardless of whether the activity was proactively proposed by the Sponsor/MAH to the HAs or requested by HAs (Fig. 10). Of the reported impact outcomes (e.g., update of PI), these changes were typically implemented within 5 to 10 years and sometimes greater than 10 years after completion of the studies.

## Discussion

To our knowledge, this is the first publication (based on a survey conducted by an industry consortium) evaluating the post-approval activities performed by pharmaceutical companies to generate data on medication use during pregnancy and lactation. From a drug development perspective, it is critical to understand how the gap of information on medication use in pregnant and lactating populations is addressed and how these data are generated and communicated to prescribers and patients. The survey results provide insights on how some Sponsors gather and leverage pregnancy and lactation information, indicating that a wide variety of post-approval activities on pregnancy and lactation have been used to assess the safety of medicinal products and to inform product labeling. These insights align with the workshops conducted with regulators, including the EMA and FDA [14, 19], where it was shown that information from various data sources could be used more effectively to aid the decision-making process by regulators, drug manufacturers, pregnant and lactating women, and healthcare professionals [14, 19].

### Limitations

While the survey provides useful information to help understand the current industry practice of safety data generation on pregnancy and lactation, the following limitations should be considered when reviewing the results. First, the possibility of limited duplication of answers exists; for example, 2 survey participants may have a partnership agreement for the development of 1 product and they both answered the survey on their shared post-approval activities. Additionally, some survey participants did not complete all parts of the survey questions possibly because activities were ongoing at the time of the survey or data may have been missing/difficult to retrieve for older studies. Perceived under- or overestimation of results may also have occurred (as the number of activities were described using ranges instead of absolute numbers), and the survey did not ask if clinically significant data added to the PI came primarily from any of the registries or from a sum of data across a variety of sources. Lastly, as the survey reflects answers from 17 pharmaceutical companies that are members of TransCelerate BioPharma, it does not reflect the activities of all Sponsors currently developing a medicinal product.

### Post-approval Activities on Pregnancy

The survey found that all 17 survey participants conducted at least 1 post-approval activity for generating data in pregnancy. The majority of these activities were requested by HAs (mostly pregnancy registries) but over 75% of the participants also proactively proposed post-approval activities on pregnancy (mostly pregnancy registries, database studies, and enhanced PV). Nearly half of the survey participants reported proactively proposing a pregnancy registry, which may reflect perceived regulatory expectations over the past 11 year timespan of the survey (January 2013–December 2023), given that newer approaches (such as database studies or enhanced PV) have gained more traction in recent years. For 59% of the survey participants, the data generated from the post-approval activities on pregnancy led to the inclusion of data in the PI. However, these changes often took from 5 years to greater than 10 years to be updated in the PI after study completion. Indeed, a review by Ayad M, et al. found that it takes 27 years to change the label from an undetermined risk in pregnancy to a defined risk [4].

The survey results demonstrate that within the last 11 years, pregnancy registries represent the most frequent post-approval activity imposed by HAs and proactively proposed by Sponsors. Based on the number of activities (that were reported as ranges), the total possible number of pregnancy registries reflected in the 17 survey responses ranged from at least 73 to at most 146 compared with at least 37 to at most 98 for enhanced PV and at least 20 to at most 70 for database studies. The benefits and limitations of using pregnancy registries to generate data on medication use during pregnancy have been intensively discussed. While registries offer the advantage of comparing different population groups in parallel and allowing prospective follow-up, numerous publications challenge their use for collecting data on pregnancy and lactation outcomes from RWD. Criticism focuses on recruitment and retention strategies as these registries can be cost- and resource-intensive, generating insufficient data that may take years to reach the medical and patient communities (as found in this survey) [12]. Another disadvantage is that the patient population in pregnancy registries may not represent the full spectrum of disease across global populations, and the sample size may limit the ability to provide a meaningful interpretation of the final results [12].

Furthermore, the quality of evidence generated depends on how the data is collected (e.g., self-reporting versus medical records, frequency of data collection) as well as the completeness of the collected data [12]. Despite these limitations, our survey found that pregnancy registries do provide information used to update the PI and may therefore be valuable in the effort to construct the safety profile of medications used in pregnancy.

The survey also found that, within the last decade, database studies have been used as post-approval activities on pregnancy, likely to complement limited data from pregnancy registries. In the survey, use of database studies resulted in significant outcomes, including PI updates (reported by 24% of participants for inclusion of data in the PI and 6% for modification of the pregnancy recommendation in the PI), removal of missing information from the product’s RMP (reported by 29% of participants), and initiation of additional post-approval activities to generate more data (reported by 12% of participants). These findings highlight the potential role of database studies in shaping risk management strategies. Database studies have the benefit of providing relatively large amounts of data compared with other types of post-approval activities and may enable generalizability to a base population [12]. As such, database studies are increasingly being requested by HAs despite their limitations, which include limited mother-infant linked data, reliance on retrospective cohort design, outcome misclassification (e.g., use diagnostic and procedure codes recorded for reimbursement purposes), exposure misclassification (e.g., prescription records do not necessarily indicate that a drug was taken and/or for how long), and a lag period for most claims databases of several months between the date of medical claim (e.g., diagnosis, prescription, clinic visit) and the date when information is available for analysis [20].

Similarly, the survey found that data collection on pregnancy through post-approval enhanced PV has been proactively proposed by several survey participants as well as requested by HAs. However, the enhanced PV reporting system can have inherent limitations, including underreporting, heterogeneity in reporting source, and lack of a comparison group [10]. Despite these limitations, data from enhanced PV can contribute to the identification of potential safety signals, which can ultimately help to better define the benefit-risk profile of a drug. Additionally, the enhanced PV approach offers advantages over pregnancy registries (such as well-documented pregnancies and outcomes) for identifying truly rare or unusual outcomes to inform the PI [21, 22].

Compared to observational data, interventional studies provide the highest-quality evidence due to the controlled environment. However, in accordance with existing guidance, they may be performed only in specific circumstances [23]. Indeed, the survey found that interventional studies, along with drug utilization studies, were the least frequent type of post-approval activities on pregnancy proposed by Sponsors or requested by HAs. The ICH has recently published a concept paper proposing a globally accepted framework and best practices to enable the inclusion and/or retention of pregnant and lactating individuals in clinical trials and might promote additional interventional studies being performed in the future [24].

### Post-approval Activities on Lactation

While the number of reported post-approval activities on lactation (ongoing or completed) was lower than the post-approval activities on pregnancy, more than 70% of the survey participants reported having at least 1 post-approval activity focused on data generation in lactation. The majority of these activities were requested by HAs and the types of post-approval activities on lactation varied from lactation registries to milk studies. In the survey, only a minority of the participants reported that post-approval activities on lactation resulted in an actual outcome. Most survey participants reported 0 impact outcomes resulting from the activities on lactation, regardless of whether the activity was proposed by the Sponsor or requested by HAs.

The lack of impact outcomes from post-approval activities on lactation, including PI changes, may reflect that most lactation studies may be ongoing or under planning, given that these are emerging methods for post-approval activities on lactation. In May 2019, the FDA released guidance on conducting clinical lactation studies [25] and since then has increasingly been requesting lactation studies to be conducted by Sponsors/MAHs [14]. Specifically, the guidance provides information on conducting lactation studies to inform medication use recommendations during lactation for inclusion in the Lactation sub-section of the US PI.

## Conclusion

There is a significant and globally recognized unmet medical need for safety data on the use of medicines during pregnancy and lactation. Therefore, it is crucial to generate both appropriate and timely data regarding medication use in this population. Post-approval safety activities can provide useful information for product labeling and help guide clinical practice. However, while regulatory guidances for post-approval activities exist, they vary depending on the ruling HA, and there remains no globally harmonized regulations or guidances on the most appropriate types of post-approval safety activities to generate critical data on medication use during pregnancy and lactation.

The TransCelerate survey of 17 pharmaceutical member companies found that most of the survey participants gathered clinically significant data from post-approval activities and that multiple types of activities on pregnancy and lactation were used to update PIs and provide critical information to the broader community of healthcare providers, patients, and caregivers. However, the majority of new medicines approved over the last years do not yet have useful pregnancy- or lactation-related information added to the PI [3, 4].

To address these challenges, it is important to seek methods and strategies to improve knowledge on the informed use of medicines for pregnant and lactating populations. Sustained efforts can help to provide relevant information for healthcare providers and pregnant and lactating women in a timely manner. Utilizing a combination of spontaneous reports, pregnancy and lactation registries, electronic healthcare data, and utilization studies can provide a robust system for post-approval safety and effectiveness monitoring drugs. Moreover, the integration of these methods with the most up-to-date signal detection and evaluation methods also can contribute to enhanced, accurate, and efficient post-approval surveillance. This comprehensive approach would allow for the detection of safety signals that may not be apparent when these methods are used in isolation. A holistic approach to allow for the rapid generation of more clinically relevant data could help to provide adequate guidance to pregnant and lactating women and their healthcare providers.

In conclusion, a multi-faceted approach to post-approval surveillance that leverages various data sources, and the latest analytical methods can provide a more complete and accurate understanding of a medication’s safety profile in the real world. This is critical for ensuring the ongoing safety of patients and the identification and effective management of risks associated with medication use during pregnancy and lactation.

## Data Availability

No datasets were generated or analysed during the current study.
